# Retinal drusen in glomerulonephritis with or without immune deposits suggest systemic complement activation in disease pathogenesis

**DOI:** 10.1038/s41598-022-12111-w

**Published:** 2022-05-17

**Authors:** P. Harraka, H. Mack, D. Colville, D. Barit, D. Langsford, T. Pianta, F. Ierino, Judy Savige

**Affiliations:** 1 University of Melbourne Department of Medicine (Melbourne Health), Parkville, VIC 3076 Australia; 2 Department of Nephrology, Northern Health, Epping, VIC 3076 Australia; 3 Department of Ophthalmology, Royal Victorian Eye and Ear Hospital, East Melbourne, VIC 3002 Australia; 4 Department of Nephrology, St Vincent’s Hospital, Fitzroy, VIC 3060 Australia

**Keywords:** Immunology, Biomarkers, Nephrology

## Abstract

Retinal drusen are characteristic of macular degeneration and complement activation, but also occur in C3, lupus and IgA nephropathy. This cross-sectional observational study compared drusen counts in different forms of glomerulonephritis. Consecutive individuals with glomerulonephritis attending a general renal or transplant clinic underwent retinal imaging with a non-mydriatic camera. Drusen were counted in deidentified images by trained graders, compared with matched hospital patients, and correlated with clinical features. Eighty-four individuals with glomerulonephritis had a mean drusen count of 10 ± 27 compared with 3 ± 8 in hospital controls (p = 0.007). Fourteen individuals with glomerulonephritis (17%) and 4 hospital controls (4/49, 8%) had increased drusen counts (≥ 10) (p = 0.20). Increased drusen counts ≥ 10 were present in 13 (13/63, 21%)  of those with glomerulonephritis and immune deposits [membranous (n = 8), antiglomerular basement membrane nephritis (n = 6), FSGS (n = 49)], and one of the 21 (5%) with glomerulonephritis without immune deposits [ANCA-associated (n = 15), minimal change disease (n = 6)]. In antibody-mediated glomerulonephritis (n = 14), mean drusen counts were 2 ± 3 in individuals with normal kidney function, 16 ± 41 with impaired function and 5 ± 7 with kidney failure . Mean counts were 24 ± 56 in individuals with glomerular IgG deposits and 1 ± 1 in those without (p = 0.76), and 23 ± 60 with complement deposits and 4 ± 8 in those without. Drusen counts were also less in immunosuppressed individuals (p = 0.049). The demonstration of retinal drusen in some forms of glomerulonephritis is consistent with systemic complement activation, and suggests that treatment targeting the complement pathways is worthwhile.

## Introduction

Retinal drusen are yellowish-white deposits that are characteristic of age-related macular degeneration and comprise oxidised membrane lipid, immunoglobulins, complement and extracellular matrix. Risk factors include genetics, age, hypertension, smoking and kidney failure^1,2^. Drusen are evident at the central macula located between the retinal pigment epithelium basal lamina and the inner collagenous layer in Bruch’s membrane^3^. Up to ten occur normally^4^. The drusen in macular degeneration may be complicated by new vessel formation, atrophy, pigmentation, and impaired vision.

The pathogenesis of drusen in macular degeneration is well understood. More than 30 genes affecting lipid metabolism, reactive oxidation, complement pathway activation, extracellular matrix integrity, apoptosis and angiogenesis have been implicated^5–7^. Drusen result from the inability to clear cell debris at the metabolically active central macula. The membrane lipid activates complement, and immunoglobulin, CRP, vitronectin and other extracellular matrix proteins are deposited locally. Nearly half the genetic risk for drusen is in *CFH* a gene that codes for an inhibitor of the alternative complement pathway^8^. Other complement genes involved include *C3, C2, CFB* and *C9*^5,6^.

Complement is also involved in many forms of glomerulonephritis (Table 1). Glomerular complement deposits are common, plasma levels are reduced in active disease, risk alleles affect complement pathway genes, and, in animal models and human disease, tissue is damaged more severely where complement is activated and less severely when complement has been depleted.

**Table 1:** Evidence for complement involvement in different forms of glomerulonephritis.

Associated with	Membranous glomerulonephritis	Anti-GBM disease	Focal and segmental glomerulosclerosis	ANCA-associated vasculitis	Minimal change glomerulonephritis
Complement genes implicated	No	No	Yes^22^	Yes	No
Altered plasma levels in active disease	Weak^23^	Yes (in some cases)^22^	Yes^24–27^	Yes^28^	No
Glomerular C3, C4d or C1q deposits	Sub-epithelial^29^	Yes^24,30^	Yes	Uncommon	Rarely
Complement activation demonstrated in glomerulonephritis	Weak^23^	Linear deposits in GBM	Yes	Yes^28^	No
A role for Complement in animal models	Yes^31^	Yes^25^	Yes^20^	Yes^32,33^	No
Clinical trials of anticomplement therapy	Yes, but variable effect^23^	Yes	No	Yes^34^	No
Previous reports of drusen	Yes (histology only)^14^	Yes^26^	No	No	No
Predominant complement pathway affected	Classical, lectin, alternative^23^	Alternative and classical	Classical	Alternative^32^	None

The demonstration of drusen in individuals with glomerulonephritis provides further evidence for a role for complement activation in glomerular disease. Drusen have been described in dense deposit disease^9,10^, lupus nephropathy^11^ and IgA glomerulonephritis^12,13^, as well as case reports in membranous and post-streptococcal glomerulonephritis^14^. There is, however, no common complement activation pathway for all these diseases. Lupus nephropathy, involves mainly the classical pathway^15^, IgA nephropathy the leptin pathway^16^ and dense deposit disease the alternative pathway^17^ but there is overlap and redundancy, and while one pathway may be important in disease initiation, another may contribute to disease progression. Drusen correlate with clinical features in at least SLE where counts are higher with longer disease duration^11^.

However it has been unclear whether drusen also occur in other forms of glomerulonephritis. These include glomerulonephritis with immune deposits (antiglomerular basement membrane (GBM) disease, and membranous glomerulonephritis, where there are glomerular IgG and C3 deposits); and Focal and Segmental glomerulosclerosis (FSGS) where there are often glomerular IgM and C3 deposits); and in glomerulonephritis where immune deposits are less common (antineutrophil cytoplasmic antibody (ANCA)-associated small vessel vasculitis, and minimal change glomerulonephritis). Both membranous nephropathy and anti-GBM disease are triggered by circulating antibodies to glomerular antigens that are also found in the retina^14,18,19^. FSGS is a histological diagnosis with diverse causes and it is not clear whether the IgM deposits initiate disease or occur secondary to podocyte damage. Recently, antibodies to podocyte proteins have been postulated to be causative^20^. ANCA-associated glomerulonephritis results from circulating antibodies but these are not deposited in the kidney and the aetiology of minimal change glomerulonephritis is unclear but not associated with glomerular complement deposits.

This study examined how often retinal drusen occur in different forms of glomerulonephritis. The similarity of the composition of drusen and glomerular immune deposits with membrane lipid, IgG, complement, CRP and extracellular proteins^14,18,19^ means that drusen may represent a model for the formation, enlargement and resorption of glomerular immune deposits, and for the development of local complications such as overlying epithelial cell atrophy.

## Patients and methods

### Study design

This was a cross-sectional observational study of individuals with glomerulonephritis recruited consecutively from a general nephrology clinic or a kidney transplant clinic at two metropolitan teaching hospitals over a 4 year period. Recruitment, data capture, and retinal photography were coordinated in a single episode and retinal images were examined for drusen by two trained graders. Control participants were age- and gender-matched hospital patients without systemic inflammatory or kidney disease recruited contemporaneously from general medical or surgical clinics.

The primary outcome was to determine if drusen occurred more often in these forms of glomerulonephritis than in controls. The secondary outcomes were to determine if drusen were associated with longer disease duration, or increased risk of kidney failure. There were no changes to the study design after its commencement and no interim analyses.

Inclusion criteria were age greater than 18 years and a renal biopsy-proven diagnosis made by a renal histopathologist or a nephrologist. Exclusion criteria were bilaterally ungradable retinal images.

This project was approved by the Northern Health Human Research Ethics Committee (HREC/16/Austin/539) and written informed consent was obtained from study participants according to the principles of the Declaration of Helsinki.

### Participants

Participants were assisted to provide a brief medical history (age, gender, disease duration, renal transplant status) and drusen risk factors (smoking, hypertension, diabetes), and their charts were reviewed for further details (eGFR, treatment with steroids, other immunosuppressants, dialysis, or transplantation).

The most recent native renal biopsy reports were reviewed where available. Immunoglobulin G or M, and C3 and C1q staining were reported as negative, 1+, 2+ or 3+.

### Measurements

#### Retinal imaging and drusen grading

All participants underwent digital retinal imaging of both optic fundi centred on the macula or optic disc of each eye with a a non-mydriatic 45° digital camera (Canon CR-DGI, Japan) in a darkened room (‘standard 2-field retinal photography’). Some were further investigated with optical coherence tomography (OCT) (Topcon, Japan or Zeiss, Germany).

Deidentified images were examined and drusen counted, independently by two trained graders, using a grid overlay corresponding to the Wisconsin Age-related maculopathy Grading System^21^. The intra-observer coefficient of correlation was 0.94.

Drusen counts were recorded from the eye with the greater number. Ten or more central drusen were considered abnormal^4^. Peripheral drusen were also counted.

Drusen size was graded by convention as small (≤ 63 µm), medium (> 63 µm, ≤ 125 µm) or large (> 125 µm) by comparison with the span of the largest arteriole or venule where they crossed the disc margin (63 µm and 125 µm respectively).

Drusen complications of overlying retinal atrophy or pigmentation were recorded by an ophthalmologist.

### Statistical analysis

This was a pilot study to determine if drusen occurred more often in these forms of glomerulonephritis than in controls, and, if so, to generate hypotheses of their clinical significance. The statistical analysis was not corrected for multiple analyses so that all possible associations were identified.

Categorical data were examined using Fisher’s exact test for dichotomous variables and Pearson Chi-square test for polytomous variables. Odds ratios and 95% confidence intervals were calculated using logistic regression analysis. Parametric data were examined using Student’s t-test and non-parametric data with the Mann–Whitney U-test. Two-sided p-values < 0.05 were considered significant and those between 0.05 and 0.10 were considered a trend. Statistical analysis was performed using SPSS v25 (IBM Corp, New York).

## Results

### Drusen in glomerulonephritis

**Figure 1:**
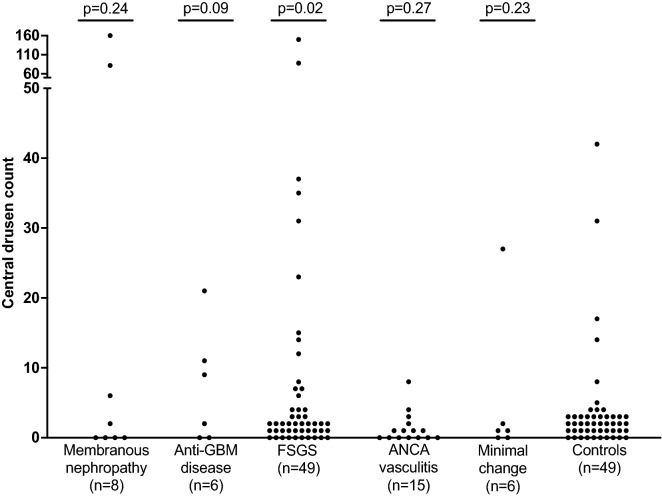
Drusen counts in different forms of glomerulonephritis and controls.

**Figure 2:**
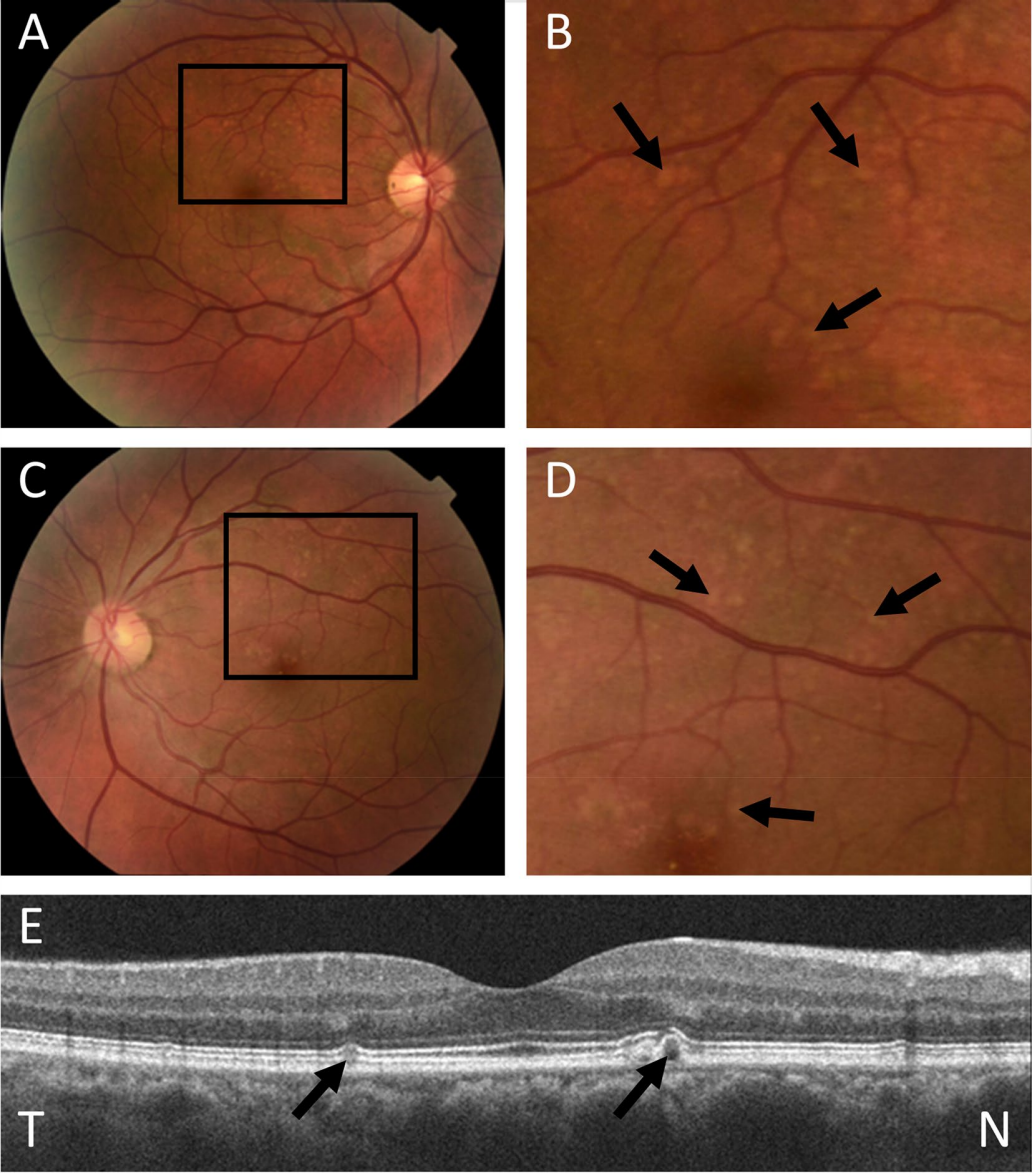
Drusen in a 50 year old man with FSGS. (**A**) Colour fundus photograph of right eye; (**B**) Magnified view demonstrating small, medium and large drusen (arrows) at the central macula; (**C**) View of left eye; (**D**) magnified view with drusen (arrows); (**E**) Optical Coherence Tomography (OCT) of the right eye demonstrating sub-retinal pigment epithelial drusen (arrows) and a break in the photoreceptor layer overlying the nasal drusen (N).

**Figure 3:**
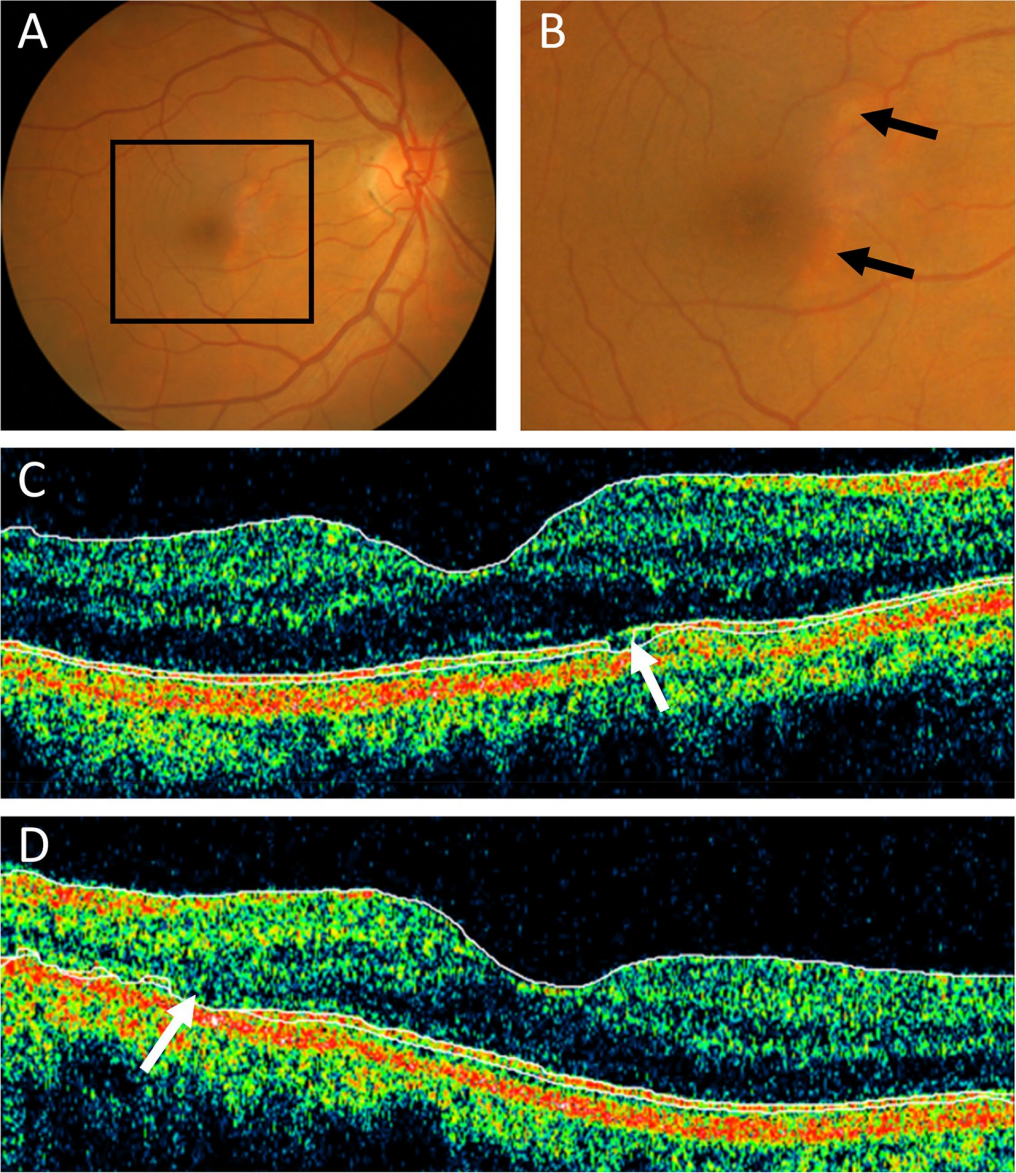
Drusen in a 60 year old man with FSGS. (**A**) Colour fundus photograph of the right eye; (**B**) Magnified view demonstrating focal pallor (arrow) in the nasal area adjacent to the fovea; (**C**) Optical Coherence Tomography (OCT) of the right eye demonstrating defects in the photoreceptor layer consistent with atrophy; and (**D**) similar defect in left eye.

Eighty-four individuals with glomerulonephritis had a mean drusen count of 10 ± 27 compared with 3 ± 8 in hospital controls (p = 0.007, Table 2, Figs. 1, 2, 3). Fourteen individuals with glomerulonephritis (17%) and four hospital controls (8%) had increased drusen counts ≥ 10 (p = 0.20). Twenty-three of those with glomerulonephritis (27%) and 10 hospital controls (20%) had medium-sized drusen (p = 0.41).

**Table 2:** Clinical features and drusen counts in different forms of glomerulonephritis.

Clinical features	All glomerulonephritis (n = 84)	Membranous glomerulo-nephritis (n = 8)	Anti-GBM disease (n = 6)	FSGS (n = 49)	ANCA-associated vasculitis (n = 15)	Minimal change glomerulo-nephritis (n = 6)	Hospital controls (n = 49)
Age (mean ± SD, years)	55 ± 15	58 ± 20	50 ± 12	55 ± 14	58 ± 17	44 ± 13	54 ± 13
**Gender, n (%)**							
Male	47 (56%)	5 (63%)	2 (33%)	29 (59%)	8 (53%)	3 (50%)	29 (59%)
Female	37 (44%)	3 (38%)	4 (67%)	20 (41%)	7 (47%)	3 (50%)	20 (41%)
**Co-morbidities, n (%)**							
Hypertension	59 (70%)	5 (63%)	4 (67%)	41 (84%)	9 (60%)	0 (0%)	22 (45%)
Diabetes	17 (20%)	2 (25%)	0 (0%)	12 (24%)	3 (20%)	0 (0%)	12 (24%)
Smoking	27 (32%)	3 (38%)	0 (0%)	15 (31%)	7 (47%)	2 (33%)	22 (45%)
Mean arterial pressure (mean ± SD, mm Hg)	96 ± 12	95 ± 13	92 ± 12	98 ± 12	93 ± 12	0 ± 9	95 ± 10
eGFR (median, IQR, ml/min/1.73 m^2^)	47 (24–77)	61 (28–86)	NA	34 (14–64)	55 (35–90)	90 (63–90)	90
**eGFR**							
Normal	12 914%)	1 (13%)	0 (0%)	3 (6%)	4 (27%)	4 (67%)	49 (100%)
Impaired	38 (45%)	6 (75%)	1 917%)	20 (41%)	9 (60%)	2 (33%)	
Kidney failure, n (%)	34 (40%)	1 (13%)	5 (83%)	26 (53%)	2 (13%)	0 (0%)	
Age at kidney failure	52 ± 15	NA	43 ± 18	52 ± 15	NA	NA	NA
Disease duration (mean ± SD)	8 ± 9	2 ± 2	9 ± 10	10 ± 9	7 ± 6	6 ± 12	NA
**Renal biopsy findings**		(n = 7)	(n = 6)	(n = 18)	(n = 5)	(n = 6)	NA
Ig staining 2+ or 3+	25 (30%)	7 (100%)	6 (100%)	11 (61%)	0 (0%)	1 (17%)	
Complement 2+ or 3+	24 (29%)	6 (86%)	6 (100%)	11 961%)	1 (20%)	0 (0%)	
Immunosuppression	46 /80 (58%)	3 (38%)	5/5 (100%)	22/47 (47%)	13/14 (93%)	3 (50%)	NA
Central drusen, mean ± SD	**10 ± 27 (p = 0.007)**	31 ± 59 (p = 0.24)	**7 ± 8 (p = 0.09)**	**9 ± 25 (p = 0.02)**	1 ± 2 (p = 0.27)	5 ± 11 (p = 0.23)	3 ± 8
≥ 10 central drusen, n (%)	14 (17%) (p = 0.20)	2 (25%) (p = 0.19)	2 (33%) (p = 0.12)	9 (18%) (p = 0.23)	0 (0%) (p = 0.57)	1 (17%) (p = 0.45)	4 (8%)
Any medium drusen, n (%)	23 (27%) (p = 0.41)	1 (13%) (p = 1.00)	0 (0%) (p = 0.58)	**20 (41%) (p = 0.048)**	2 (13%) (p = 0.72)	0 (0%) (p = 0.58)	10 (20%)

Significant values are in bold.

*CI* confidence interval, *eGFR* estimated glomerular filtration rate mL/min/1.73 m^2^, *IQR* interquartile range, *SD* standard deviation, *NA* not applicable.

### Drusen in glomerulonephritis with immune deposits

#### Membranous glomerulonephritis

Eight individuals were examined, with a mean age of 58 ± 20 years and including 5 males (63%) (Table 2). One had a normal eGFR, 6 had impaired kidney function, and one had kidney failure. Their mean disease duration was 2 ± 2 years, 7 had IgG staining and 6 had Complement staining in their renal biopsies. Three (38%) were currently immunosuppressed.

Their mean central drusen count was 31 ± 59 (p = 0.24), and the number with drusen ≥ 10 or with medium-sized drusen were also not different from controls (p = 0.19, p = 1.00, respectively).

#### AntiGBM disease

Six individuals were examined with a mean age of 50 ± 12 years and including 2 males (33%) (Table 2). One had impaired kidney function and five had kidney failure. Their mean disease duration was 9 ± 10 years. All were currently immunosuppressed.

Their mean central drusen count demonstrated a trend to greater than in controls (7 ± 8 and 3 ± 8 respectively, p = 0.09), but the number with drusen ≥ 10 or with medium-sized drusen were not different from controls.

When both membranous and antiGBM disease were considered together (antibody-mediated glomerulonephritis) total drusen counts were increased more than in controls (38 ± 87 and 1 ± 2 respectively p = 0.09) and 5 had drusen ≥ 10 (p = 0.04, OR 15.6, 95% CI 0.77–318). (Table 3). There was no difference in the likelihood of medium-sized or large drusen nor of pigmentation or atrophy (p all NS).

**Table 3:** Clinical features and drusen in antibody-mediated glomerulonephritis and controls.

Clinical characteristics	Antibody-mediated glomerulonephritis (n = 14)	Controls (n = 13)	OR (95% CI), p-value
Age (mean ± SD, years)	54 ± 17	50 ± 13	p = 0.46
**Gender, n (%)**			
Male	7 (50%)	7 (54%)	0.86 (0.19–3.89), 1.00
Female	7 (50%)	6 (46%)	
**Co-morbidities, n (%)**			
Hypertension	9 (64%)	4 (31%)	4.05 (0.81–20.2), 0.13
Diabetes	2 (14%)	1 (8%)	2.00 (0.16–25.1), 1.00
Smoking history	3/13 (23%)	7 (54%)	0.26 (0.05–1.39), 0.23
Mean arterial pressure (mean ± SD, in mmHg)	94 ± 12	94 ± 10	p = 0.83
Current immunosuppression, n (%)	8/13 (62%)	0 (0%)	NA
**Kidney function**			
Normal eGFR, n (%)	1 (7%)	NA	NA
Impaired eGFR, n (%)	7 (50%)		
ESKF, n (%)	6 (43%)		
Transplant, n (%)	4 (29%)		
Age at kidney failure (mean ± SD)	46 ± 17		
Kidney disease duration (mean ± SD, in years)	(n = 13) 5 ± 8	NA	NA
**Renal biopsies (n = 7)**			
IgG staining	7 (100%)	NA	NA
Complement staining	6 (86%)		
**Drusen count**			
Central, mean ± SD	21 ± 45	1 ± 2	p = **0.09**
≥ 10, n (%)	4 (29%)	0 (0%)	11.6 (0.56–240), **0.098**
Peripheral, mean ± SD	17 ± 43	1 ± 1	p = 0.30
≥ 10, n (%)	5 (36%)	0 (0%)	15.6 (0.77–318), **0.04**
Total, mean ± SD	38 ± 87	2 ± 3	p = 0.20
≥ 10, n (%)	5 (36%)	0 (0%)	15.6 (0.77–318), **0.04**
Drusen in ≥ 4 macular quadrants, n (%)	7 (50%)	0 (0%)	**27.0 (1.35–542), 0.006**
**Drusen size, n (%)**			
Any medium drusen	1 (7%)	2 (15%)	0.42 (0.03–5.32), 0.60
Any large drusen	1 (7%)	0 (0%)	3.00 (0.11–80.4), 1.00
Pigmentation, n (%)	0 (0%)	0 (0%)	NA
Atrophy, n (%)	0 (0%)	0 (0%)	NA

Significant values are in bold.

Antibody-mediated glomerulonephritis includes membranous nephropathy and anti-GBM disease. *CI* confidence interval, *eGFR* estimated glomerular filtration rate mL/min/1.73 m^2^, *IQR* interquartile range, *NA *not applicable.

#### Focal and segmental glomerulosclerosis

Forty-nine individuals with FSGS were examined, with a mean age of 55 ± 14 years and including 29 males. FSGS appeared to be primary in two (4%) and secondary to nephrectomy (2, 4%), reflux nephropathy (1, 2%) or probable viral disease (6, 21%). Likely familial disease was present in 10 (20%) based on the presence of another affected family member or extra-renal features in 7 (14%) (epilepsy, congenital heart defect, sensorineural hearing loss, intellectual disability or abnormal ears, or digits).

Three individuals with FSGS had a normal eGFR, 20 had impaired kidney function, and 26 had kidney failure. Their mean disease duration was 10 ± 9 years, and of the 18 kidney biopsies, 11 had IgM and complement staining. Twenty-two (47%) were currently immunosuppressed. None of those with a kidney transplant had recurrent glomerulonephritis.

Their mean central drusen count was 9 ± 25 which was more than in controls (p = 0.02), the number with drusen ≥ 10 was not different from controls (p = 0.23) but medium-sized drusen were more common (OR 2.69 CI 1.10–6.61, p = 0.048) (Table 4).

**Table 4:** Clinical features and drusen in Focal and Segmental Glomerulosclerosis and controls.

Characteristics	FSGS (n = 49)	Controls (n = 49)	OR (95% CI), p-value
Age, mean ± SD, in years	55 ± 14	54 ± 13	p = 0.78
**Gender, n (%)**			
Male	29 (59%)	29 (59%)	1.00 (0.45–2.24), 1.00
Female	20 (41%)	20 (41%)	
**Co-morbidities, n (%)**			
Hypertension	41 (84%)	22 (45%)	**6.29 (2.45–16.2), < 0.001**
Diabetes	12 (24%)	12 (24%)	1.00 (0.40–2.51), 1.00
Smoking history	15/48 (31%)	22 (45%)	0.56 (0.24–1.28), 0.21
Mean arterial pressure (mean ± SD in mmHg)	98 ± 12	95 ± 10	p = 0.47
**FSGS classification, n (%)**			
Primary	2 (4%)	NA	NA
Secondary—post-nephrectomy, viral	12 (24%)		
Genetic	16 (33%)		
Current immunosuppression, n (%)	22 (47%)	0 (0%)	NA
**Kidney function**			
Normal eGFR, n (%)	3 (6%)	NA	NA
Impaired eGFR, n (%)	20 (41%)		
Kidney failure, n (%)	26 (53%)		
Transplant, n (%)	16 (33%)		
Age at kidney failure, mean ± SD, years	52 ± 15		
Kidney disease duration in years, mean ± SD (n = 38)	10 ± 9	NA	
**Renal biopsies (n = 18)**			
IgM staining			
3+ or 2+	11 (61%)	NA	NA
1+ or none	7 (39%)		
C3 staining			
3+ or 2+	8 (44%)		
1+ or none	10 (56%)		
Mean central drusen ± SD	9 ± 25	3 ± 8	**p = 0.02**
≥ 10 drusen in worse eye, n (%)	9 (18%)	4 (8%)	2.53 (0.72–8.86), 0.23
**Drusen size, n (%)**			
Any medium drusen	20 (41%)	10 (20%)	**2.69 (1.10–6.61), 0.048**
Any large drusen	4 (8%)	3 (6%)	1.36 (0.29–6.44), 1.00
Pigmentation, n (%)	3 (6%)	0 (0%)	7.45 (0.37–148), 0.24
Atrophy, n (%)	9 (18%)	0 (0%)	**23 (1.31–411), 0.003**

Significant values are in bold.

*CI* confidence interval, *eGFR* estimated glomerular filtration rate mL/min/1.73 m^2^, *IQR* interquartile range, *SD* standard deviation, *NA* not applicable.

Retinal atrophy was present in 9 individuals with FSGS (9, 18%) which was more often than in controls (0, 0%) (OR 23 CI 1.31–411, p = 0.003). Abnormal pigmentation occurred in three (3, 6%) with FSGS but no controls (OR 7.45 CI 0.37–148, p = 0.24). Atrophy was more common (p = 0.003).

Four individuals with FSGS underwent optical coherence tomography. One of these, a 50 year old man, had bilateral drusen present in the sub-retinal pigment epithelial space. Another, a 60 year old man, had focal outer retinal atrophy but no drusen (Fig. 2) and the other two had normal OCT examinations.

Drusen counts ≥ 10 were demonstrated in individuals with FSGS from apparently diverse causes, including secondary to nephron loss or viral infections, or with likely genetic causes (5, 10%). Neither of the two with primary FSGS had drusen.

Higher drusen counts, counts ≥ 10 and larger drusen were not associated with age > 60, sex, drusen risk factors, mean arterial pressure, kidney function, age at kidney failure, disease duration, or immune staining (p all NS), except that drusen were larger in individuals with FSGS and diabetes (8, 67%; 12, 32%; p = 0.048) (Supplementary Table 1).

Eleven individuals (7M, 4F, mean age 49 ± 15 years) with FSGS underwent retinal imaging a second time after a mean of 6 ± 4 years. Their initial median drusen count was 2 (range 0–246) with a follow up median drusen count of 2 (0–103). At the second examination, counts were unchanged in 4, increased in 2 and decreased in 5.

### Drusen in glomerulonephritis without immune deposits

#### ANCA-associated vasculitis

Fifteen individuals were examined, with a mean age of 58 ± 17 years and including 8 males (Table 2). Four had a normal eGFR, 9 had impaired kidney function and two had kidney failure. Their mean disease duration was 7 ± 6 years, and none had IgG but one had complement staining in their kidney biopsies. Thirteen were currently immunosuppressed.

Their mean central drusen count was 1 ± 2 which was not different from controls (p = 0.27), and the number with drusen ≥ 10 (p = 0.57) or with medium-sized drusen (p = 0.72) were also not different.

#### Minimal change glomerulonephritis

Six individuals were examined, with a mean age of 44 ± 13 years and including 3 males (Table 2). Four had a normal eGFR, and 2 had impaired kidney function. Their mean disease duration was 6 ± 12 years and one had IgG staining but no complement in their kidney biopsies. Three were currently immunosuppressed.

Their mean central drusen count was 5 ± 11, which was not different from controls (p = 0.23), and the number with drusen ≥ 10 (p = 0.45) or with medium-sized drusen (p = 0.58) were also not different.

### Comparison of glomerulonephritis with and without immune deposits

Mean drusen count in antibody-mediated glomerulonephritis with immune deposits (membranous, antiGBM disease) was 21 ± 45 and 2 ± 6 in glomerulonephritis without immune deposits (ANCA-associated vasculitis, minimal change glomerulonephritis) (p = 0.28). Counts ≥ 10, medium-sized or large drusen, and pigmentation and atrophy were not also different in both groups (Table 5).

**Table 5:** Clinical features and drusen in antibody-mediated glomerulonephritis and glomerulonephritis without immune deposits.

Clinical characteristics	Antibody-mediated glomerulo-nephritis (n = 14)	Glomerulo-nephritis without immune deposits (n = 21)	OR (95% CI), p-value
Age in years, mean ± SD	54 ± 17	54 ± 17	p = 0.95
**Gender, n (%)**			
Male	7 (50%)	11 (52%)	0.91 (0.24–3.52), 1.00
Female	7 (50%)	10 (48%)	
**Co-morbidities, n (%)**			
Hypertension	9 (64%)	9 (43%)	2.40 (0.60–9.67), 0.31
Diabetes	2 (14%)	3 (14%)	1.00 (0.15–6.91), 1.00
Smoking history	3/13 (23%)	9 (43%)	0.40 (0.09–1.89), 0.29
**Mean arterial pressure in mmHg**			
Mean ± SD	94 ± 12	92 ± 11	p = 0.65
Immunosuppression, n (%)	8/13 (62%)	16/20 (80%)	0.40 (0.08–1.91), 0.43
**Kidney function (no transplant)**			
eGFR, median (IQR)	56 (20 to 83)	61 (37 to 90)	p = 0.26
**Kidney function**			
Normal eGFR, n (%)	1 (7%)	8 (38%)	
Impaired eGFR, n (%)	7 (50%)	11 (52%)	**p = 0.03**
ESKF, n (%)	6 (43%)	2 (10%)	
Transplant, n (%)	4 (29%)	0 (0%)	18.4 (0.91–375), **0.02**
Age at ESKF, mean ± SD	46 ± 17	NA	**NA**
Kidney disease duration in years Mean ± SD	(n = 13) 5 ± 8	6 ± 8	p = 0.53
**Renal biopsies**	n = 7	n = 11	
IgG staining	7 (100%)	1 (9%)	**105 (3.74–2948), 0.0003**
Complement staining	6 (86%)	1 (9%)	**60.0 (3.14–1147), 0.003**
Mean central drusen ± SD	21 ± 45	2 ± 6	p = 0.28
≥ 10, n (%)	4 (29%)	1 (5%)	8.00 (0.79–81.3), 0.13
Drusen in ≥ 4 macular zones, n (%)	7 (50%)	5 (24%)	3.20 (0.75–13.7), 0.15
**Drusen size, n (%)**			
Any medium or large drusen	1 (7%)	2 (10%)	0.73 (0.06–8.92), 1.00
Pigmentation, n (%)	0 (0%)	0 (0%)	NA
Atrophy, n (%)	0 (0%)	0 (0%)	**NA**

Significant values are in bold.

Antibody-mediated glomerulonephritis included membranous nephropathy and anti-GBM disease. Glomerulonephritis without immune deposits included ANCA-associated vasculitis and minimal change disease. *eGFR* estimated glomerular filtration rate mL/min/1.73 m^2^, *CI* confidence interval, *IQR* interquartile range, *OR* odds ratio, *SD* standard deviation, *NA* not applicable.

In individuals with antibody-mediated glomerulonephritis with immune deposits (membranous, antiGBM disease) and glomerulonephritis without immune deposits (ANCA-associated vasculitis, minimal change glomerulonephritis), central drusen counts were increased in those  > 60 years (p = 0.02) and counts were less with immunosuppression (p = 0.049) (Supplementary Table 2). Counts ≥ 10 and medium-large drusen were not associated with age > 60, gender, drusen risk factors, mean arterial pressure, kidney function, age at kidney failure, disease duration or immune staining (p all NS).

## Discussion

Individuals with different types of glomerulonephritis had more retinal drusen than hospital controls. In particular, drusen counts were increased in antiGBM disease and FSGS which are both associated with glomerular immune deposits but not in ANCA-associated vasculitis or minimal change glomerulonephritis where there are few or no glomerular deposits. In summary, drusen counts ≥ 10 were found in about 20% of individuals with glomerulonephritis with immune deposits but only 6% of those without, as well as in 8% of hospital controls.

Thus drusen are increased in different types of glomerulonephritis but especially those with glomerular immune deposits. Drusen were not however present in everyone with any type of glomerulonephritis and sometimes also occurred in glomerulonephritis without immune deposits. This variability may be explained by some diseases activating complement less or by the study’s cross-sectional nature meaning that drusen were counted at different disease stages including inactive disease. Antibody-mediated glomerulonephritis which included membranous and antiGBM nephropathy, and FSGS were examined separately although all were potentially associated with immune deposits. This was because FSGS has diverse causes and any overall association with drusen may have been masked by the inclusion of a predominant type where drusen did not occur.

The likelihood of drusen ≥ 10 has been demonstrated previously to vary from 20% in IgA disease and 20–40% in SLE to nearly everyone with dense deposit disease^9–13^. The drusen counts observed here in membranous, antiGBM disease and FSGS were consistent with the observations in IgA disease and SLE. These counts were still likely to be underestimates because retinal imaging is relatively insensitive for drusen detection^11^. Even if the actual numbers were higher, our conclusions still hold true for drusen that are visible with retinal imaging.

Drusen were concentrated in the central macula which is where there are most photoreceptors with their high energy expenditure. Oxidised lipids and immune complexes are more likely to be trapped here and presumably trigger uncontrolled complement activation predisposing to drusen formation. Peripheral drusen appeared less often and more randomly in glomerulonephritis.

Drusen were not usually larger in glomerulonephritis except in individuals with FSGS. However drusen size in these cases was not related to disease duration or risk of kidney failure. Size may instead have been from to increased complement activation or more persistent activation.

The drusen complications of retinal atrophy and pigmentation were also noted. Atrophy is the end-result of drusen resorption beneath the retinal pigment epithelium and was increased in FSGS. Retinal atrophy also occurs with poorly controlled hypertension which is more common in FSGS and in other forms of glomerulonephritis.

The drusen in glomerulonephritis were unlikely to be due to coincidental macular degeneration because they were demonstrated from a younger age, were located at different level in the retina (between the retinal pigment epithelial cells and Bruch’s membrane, in contrast to beneath Bruch’s membrane), were smaller, did not increase substantially in number or size over time, and were not associated with visual loss or other complications. The difference in location may be due to different pathogeneses. In macular degeneration the apoptotic retinal pigment epithelial cell lipid activates complement and other circulating immune molecules adhere. In glomerulonephritis the drusen are likely to result from the deposition of immune complexes in the central retina. The drusen in glomerulonephritis were also unlikely to be caused by another retinal disease because these had been excluded on examination by an ophthalmologist.

This study was designed to determine if drusen were increased in different forms of glomerulonephritis or were clinically significant. It demonstrated that drusen counts were lower after immunosuppression. Immunosuppression is used in the initial management of some forms of glomerulonephritis and after kidney transplantation. It is unlikely to affect drusen development directly but reduces antibody formation and disease activity which both potentially lessen drusen risk.

There also appeared to be more drusen in individuals with impaired kidney function or with antibody and complement staining of the glomerular deposits, although these results did not reach significance. In general, the individual disease cohorts were too small and the drusen counts too variable to demonstrate any correlation.

There was no association of drusen counts with the macular degeneration risk factors of sex and smoking. These results may have been skewed by some immune-mediated forms of glomerulonephritis such as anti-GBM disease being more common in males and the practice of not accepting patients onto a kidney transplant programme until they stopped smoking. The traditional risk factors for macular degeneration may be less important in glomerulonephritis where drusen development depends more on retinal immunoglobulin deposition than on apoptosis for local complement activation.

Direct measurement of complement levels was not possible in this study because many patients were recruited years after their initial presentation and often after kidney transplantation. Complement levels are reduced in only a few forms of active glomerulonephritis such as post-streptococcal and lupus nephritis which were not examined here. We did not attempt to correlate drusen with the genetic risk alleles associated with macular degeneration because these are typically normal variants that required much larger cohorts to demonstrate an effect.

The strengths of this study were the diverse forms of glomerulonephritis examined, the reproducible methods for counting drusen, and the use of matched and well-characterised controls. The study’s limitations were its cross-sectional nature where retinal images were recorded at different disease stages, the relatively small cohorts, and the use of colour fundus images that only detected larger drusen.

Increased drusen counts occurred in all forms of glomerulonephritis associated with glomerular immune deposits and less often in those without deposits, such as ANCA-associated vasculitis and minimal change glomerulonephritis. The demonstration of drusen in many forms of glomerulonephritis is consistent with the major role for complement activation demonstrated by retinal histology and GWAS. However unlike macular degeneration where drusen are associated with alternative pathway activation, drusen in glomerulonephritis are associated with diverse complement activation pathways. It is unclear why only some individuals with any form of glomerulonephritis develop drusen. These observations also suggest that treatments targeting complement may be useful in these diseases. Future studies should confirm the association with clinical features suggested here and investigate any correlation with disease activity and treatment.

## Supplementary Information

Supplementary Tables.

## Data Availability

The datasets generated and analysed during the current study are not publicly available because the primary author wishes to undertake further analyses but they are available from the corresponding author on reasonable request.
