# Supplementary material for: Retinal drusen in glomerulonephritis with or without immune deposits suggest systemic complement activation in disease pathogenesis
Source: Sci Rep. 2022 May 17;12:8234. doi: 10.1038/s41598-022-12111-w (PMC9114393; doi:10.1038/s41598-022-12111-w)
Supplement: Supplementary file 1 — Supplementary Tables. [file 41598_2022_12111_MOESM1_ESM.pdf]

Supplemental List of Tables

Suppl Table 1: Clinical associations of drusen in Focal and Segmental Glomerulosclerosis

Suppl Table 2: Clinical associations of drusen in antibody-mediated glomerulonephritis and glomerulonephritis without immune deposits

Suppl Table 1: Clinical associations of drusen in Focal and Segmental Glomerulosclerosis

Clinical features	Central drusen, mean ± SD	p-value	≥10 drusen centrally, n (%)	OR (95% CI), p-value	Medium or large drusen, n (%)	OR (95% CI), p-value
Age in years						
≥60 (n=19)	11 ± 35	0.41	2 (11%)	0.39 (0.07 to 2.10), 0.45	10 (53%)	2.22 (0.68 to 7.22), 0.24
<60 (n=30)	8 ± 18		7 (23%)		10 (33%)	
Age in years						
≥40 (n=43)	10 ± 27	0.62	8 (19%)	1.14 (0.12 to 11.2), 1.00	18 (42%)	1.44 (0.24 to 8.73), 1.00
<40 (n=6)	3 ± 5		1 (17%)		2 (33%)	
Sex						
Male (n=29)	7 ± 18	0.40	4 (14%)	0.48 (0.11 to 2.07), 0.46	13 (45%)	1.51 (0.47 to 4.88), 0.56
Female (n=20)	13 ± 33		5 (25%)		7 (35%)	
Co-morbidities						
Hypertension (n=41)	10 ± 27	0.70	7 (17%)	0.62 (0.10 to 3.72), 0.63	16 (39%)	0.64 (0.14 to 2.93), 0.70
No hypertension (n=8)	7 ± 12		2 (25%)		4 (50%)	
Diabetes (n=12)	16 ± 42	0.15	2 (17%)	0.86 (0.15 to 4.82), 1.00	8 (67%)	**4.17 (1.04 to 16.6), 0.048**
No Diabetes (n=37)	7 ± 17		7 (19%)		12 (32%)	
Smoking history (n=15)	10 ± 14	0.16	5 (33%)	3.63 (0.81 to 16.2), 0.12	6 (40%)	0.90 (0.26 to 3.13), 1.00
No smoking (n=33)	10 ± 30		4 (12%)		14 (42%)	
Mean arterial pressure						
≥107mmHg (n=11)	6 ± 11	0.63	2 (18%)	1.04 (0.18 to 6.07), 1.00	6 (55%)	2.20 (0.55 to 8.74), 0.30
<107mmHg (n=34)	11 ± 30		6 (18%)		12 (35%)	
Kidney function						
Normal eGFR (n=3)	1 ± 2	0.26	0 (0%)	p=0.49	0 (0%)	p=0.33
Impaired eGFR (n=20)	11 ± 21		5 (25%)		9 (45%)	
Kidney failure (n=26)	9 ± 30		4 (15%)		11 (42%)	
Age at kidney failure in years						
≥40 (n=19)	11 ± 35	0.47	3 (16%)	1.06 (0.04 to 27.3), 1.00	9 (47%)	4.52 (0.19 to 107), 0.49
<40 (n=2)	2 ± 1		0 (0%)		0 (0%)	
Disease duration in years						
≥10 (n=18)	11 ± 22	0.74	4 (22%)	1.81 (0.35 to 9.41), 0.68	6 (33%)	0.60 (0.17 to 2.18), 0.53
<10 (n=22)	5 ± 9		3 (14%)		10 (45%)	
Immunosuppression						
Yes (n=22)	4 ± 8	0.26	3 (14%)	0.50 (0.11 to 2.30), 0.47	8 (36%)	0.86 (0.26 to 2.79), 1.00
No (n=25)	15 ± 34		6 (24%)		10 (40%)	
Capillary loop deposits (n=18)						
IgM staining						
Present (n=11)	12 ± 26	0.54	2 (18%)	0.56 (0.06 to 5.24), 1.00	2 (18%)	0.17 (0.02 to 1.42), 0.14
Absent (n=7)	8 ± 13		2 (29%)		4 (57%)	
Complement staining						
Present (n=11)	12 ± 26	0.54	2 (18%)	0.56 (0.06 to 5.24), 1.00	2 (18%)	0.17 (0.02 to 1.42), 0.14
Absent (n=7)	8 ± 13		2 (29%)		4 (57%)	

eGFR = estimated glomerular filtration rate mL/min/1.73m^2, IQR = interquartile range, CI = confidence interval, OR = odds ratio, SD = standard deviation, NA not applicable

Suppl Table 2: Clinical associations of drusen in antibody-mediated glomerulonephritis and glomerulonephritis without immune deposits

Clinical features	Central drusen, mean ± SD	p-value	≥10 drusen centrally, n (%)	OR (95% CI), p-value	Medium or large drusen, n (%)	OR (95% CI), p-value
Age in years						
≥60 (n=14)	12 ± 43	**0.02**	1 (7%)	0.33 (0.03 to 3.28), 0.63	2 (14%)	3.33 (0.27 to 40.8), 0.55
<60 (n=21)	9 ± 18		4 (19%)		1 (5%)	
Age in years						
≥40 (n=29)	12 ± 33	0.22	5 (17%)	2.92 (0.14 to 59.9), 0.56	3 (10%)	1.72 (0.08 to 37.5), 1.00
<40 (n=6)	1 ± 1		0 (0%)		0 (0%)	
Sex						
Male (n=18)	8 ± 20	0.64	3 (17%)	1.50 (0.22 to 10.3), 1.00	0 (0%)	0.11 (0.01 to 2.35), 0.10
Female (n=17)	12 ± 38		2 (12%)		3 (18%)	
Co-morbidities						
Hypertension (n=18)	16 ± 41	0.41	3 (17%)	1.50 (0.22 to 10.3), 1.00	2 (11%)	2.00 (0.16 to 24.3), 1.00
No hypertension (n=17)	4 ± 7		2 (12%)		1 (6%)	
Diabetes (n=5)	17 ± 37	0.54	1 (20%)	1.63 (0.14 to 18.5), 0.56	1 (20%)	3.50 (0.26 to 48.0), 0.38
No Diabetes (n=30)	9 ± 29		4 (13%)		2 (7%)	
Smoking history (n=12)	21 ± 50	0.29	2 (17%)	2.00 (0.24 to 16.4), 0.60	1 (8%)	0.91 (0.07 to 11.2), 1.00
No smoking (n=22)	4 ± 7		2 (9%)		2 (9%)	
Mean arterial pressure						
≥107mmHg (n=3)	2 ± 2	0.95	0 (0%)	1.00 (0.04 to 23.8), 1.00	1 (33%)	13.0 (0.57 to 295), 0.19
<107mmHg (n=27)	9 ± 31		3 (11%)		1 (4%)	
Kidney function						
Normal eGFR (n=9)	2 ± 3	0.94	0 (0%)	p=0.29	1 (11%)	p=0.62
Impaired eGFR (n=18)	16 ± 41		4 (22%)		2 (11%)	
Kidney failure (n=8)	5 ± 7		1 (13%)		0 (0%)	
Age at Kidney failure in years						
≥40 (n=6)	6 ± 8	0.64	1 (17%)	1.36 (0.04 to 46.7), 1.00	0 (0%)	NA
<40 (n=2)	1 ± 1		0 (0%)		0 (0%)	
Disease duration in years						
≥10 (n=9)	1 ± 3	0.38	0 (0%)	0.25 (0.01 to 5.15), 0.55	0 (0%)	0.34 (0.02 to 7.21), 0.55
<10 (n=25)	10 ± 32		4 (16%)		3 (12%)	
Immunosuppression						
Yes (n=24)	3 ± 7	**0.049**	2 (8%)	0.32 (0.04 to 2.70), 0.30	2 (8%)	0.73 (0.06 to 9.16), 1.00
No (n=9)	28 ± 56		2 (22%)		1 (11%)	
Capillary loop deposits (n=18)						
IgG staining						
Present (n=8)	24 ± 56	0.76	2 (25%)	8.08 (0.33 to 196), 0.18	1 (13%)	1.29 (0.07 to 24.4), 1.00
Absent (n=10)	1 ± 1		0 (0%)		1 (10%)	
Complement staining						
Present (n=7)	23 ± 60	0.15	1 (14%)	1.67 (0.09 to 31.9), 1.00	1 (14%)	1.67 (0.09 to 31.9), 1.00
Absent (n=11)	4 ± 8		1 (9%)		1 (9%)	

eGFR = estimated glomerular filtration rate mL/min/1.73m^2, CI = confidence interval, IQR = interquartile range, OR = odds ratio, SD = standard deviation, NA not applicable
